# Carbon monoxide suicide by charcoal-burning: a case report and review of the literature

**DOI:** 10.11604/pamj.2021.40.190.22783

**Published:** 2021-11-30

**Authors:** Youssef Nouma

**Affiliations:** 1 Forensic Department, Teaching Hospital Habib Bourguiba, Medenine, Tunisia

**Keywords:** Autopsy, poisoning, carbon monoxide, charcoal, suicide, case report

## Abstract

Charcoal burning (CB) suicide is an old method that revived and has clearly become prevalent across several countries, as considered “soft” and “painless”. This alarming spread is induced by the contagious effect of suicidal behaviors. Internet and suicide web forums may play, unfortunately, a crucial role in promoting this suicide's method. In this paper, we present the first documented case of CB suicide in Tunisia and probably in all African countries. Thereupon, we report a case of a young man suicide and we detail the forensic investigation findings. In order to prevent a potential epidemic spread, this form of suicide should require further attention not only in regions where it is already recognized but also in countries where it is not yet prevalent.

## Introduction

Carbon monoxide (CO) poisoning remains associated with high rates of mortality and morbidity throughout the world. It may be the cause of more than 50% of fatal poisonings reported in many countries [1]. The odorless and non-irritating characteristics of CO explain the occult poisoning, not only in accidental cases where a large number of victims is simultaneously affected but also in cases of deliberate intoxication.

In fact, suicide by CO poisoning has a long history all over the world. In Europe before the 1990´s, the majority of CO poisoning was accidental, the proportion of suicides was far less than five per cent [2]. Most suicide cases were caused by inhalation of domestic coal gas supply or automobile exhaust especially in an enclosed space like a garage. Following the widespread detoxification of home gas and the introduction of automobile catalytic converter, CO suicide rate has even more dropped [3]. However, new variants of CO deliberate poisoning have emerged, such as the chemical induction of CO or charcoal burning in a confined space [4]. This latter method of suicide has occurred for decades in Eastern Asia, mainly in the context of suicide pacts [4]. Since the information on suicide has become easily accessible on the internet, CB suicide is spread around the world as a result of imitation [5].

## Patient and observation

**Figure 1:**
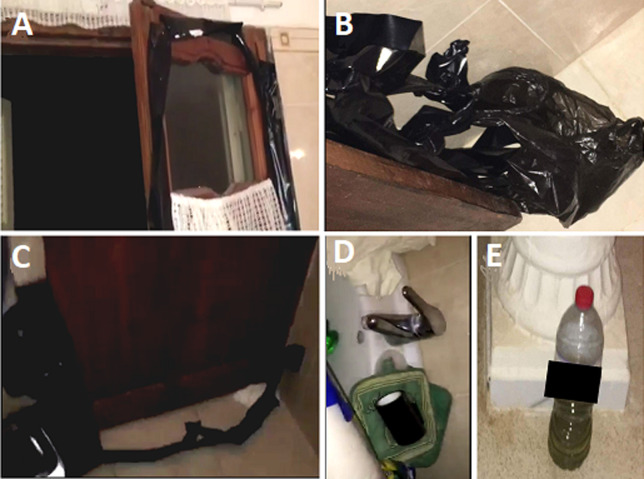
death scene inspection, A) presence of black plastic bands taped to the edges of the window and; B,C) the door; D) the adhesive tape used and; E) a plastic bottle containing a petroleum product

**Figure 2:**
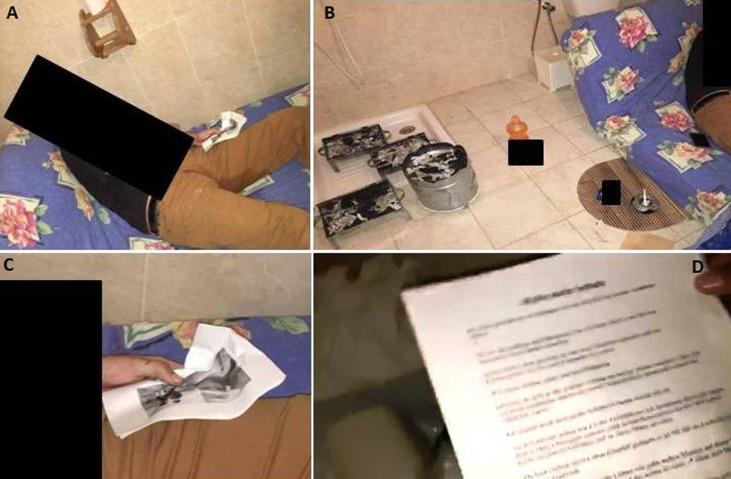
death scene inspection, A) general view and position of the deceased; B) the close environment and the barbecue grills; C) the printed photo captured by the left hand and; D) a suicide note

A 28-year-old deceased man was discovered locked inside the bathroom of a rented house. He was a tourist that arrived at the airport the day before and rented a house by the sea. The death scene investigation (Figure 1, Figure 2) found the presence of black plastic bands taped to the inner faces of the window and the door's edges clogging thereby the air inlet. Even the keyhole of the bathroom door was clogged with paper and ribbon tape. Inside, the deceased body was semi-lying on a mattress next to the bathroom wall and holding a paper in his left hand on which was printed his picture accompanied by a young woman. Just below his feet, there was a suicide note in which he indicates that betrayal and a relationship breakup are the reasons behind his suicide. On his right side, there was a cell phone closed by electric charge drop. Even further to the right, we found three barbecue grills and a brazier. It was hardly used and it contained ashes with charred coal residue. However, we did not find any potentially dangerous substances, except for one plastic bottle containing a petroleum product used to make fire.

The police investigation confirmed that the deceased arrived alone at the airport. The neighborhood merchant avows that the deceased had purchased barbecue grills, charcoal, soda drink and one liter of petroleum product to prepare a barbecue dinner. The surveillance camera in front of the house did not record any visitors. The examination of the deceased's phone eliminated recent suspect phone contacts. The review of the telephone browser history revealed that he was searching on the internet for terms like «easy suicide» and consulting a web-forum about suicide. In this regard, a detailed description of an American famous singer suicide by CO poisoning using charcoal burning in the bathroom was exposed.

**Figure 3:**
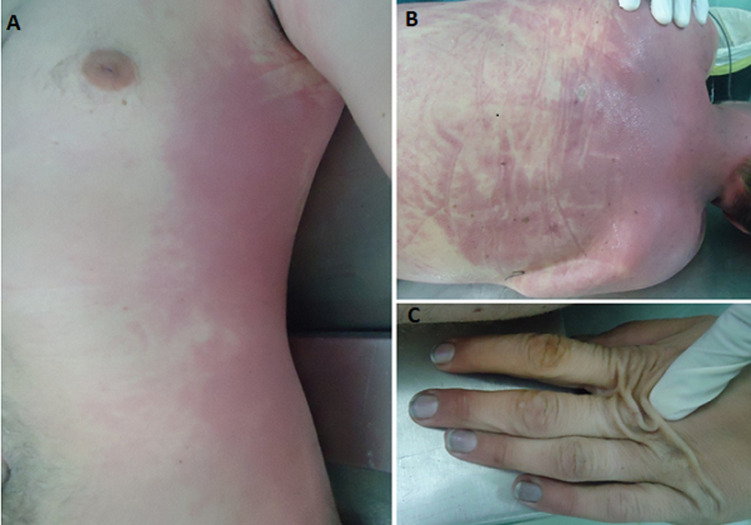
the external examination findings, A,B) cherry red livor mortis on posterior and left sides; C) blackish incrustations under the fingernails of hands

**Figure 4:**
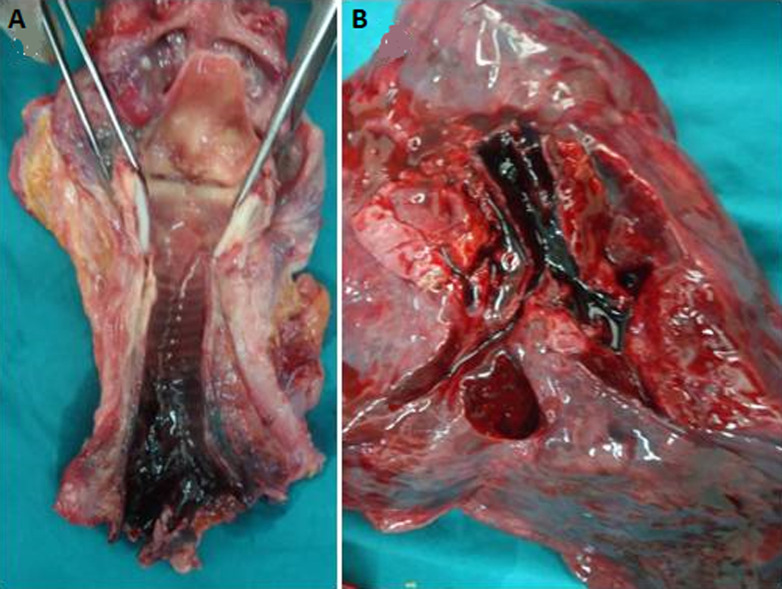
autopsy findings, A,B) black soot in the airways and cherry red coloration

The external examination carried out during autopsy, 48 hours later, revealed the beginning of putrefaction with rigor mortis resolution, green discoloration at the right flank, cherry red livor mortis on posterior and left sides (Figure 3(A,B)), and particularly blackish incrustations under the fingernails of hands (Figure 3C). Autopsy showed cherry-red coloration of blood and muscles with black soot in the airways (Figure 4), cerebral and pulmonary edema (brain weight=1660g, lungs weight=1180/1025g) and greyish liquid in the stomach without particular odor. Toxicological analyses performed five days later, on samples of peripheral and cardiac blood, urine and gastric contents indicated the only presence of carboxyhemoglobin (COHb) in the blood at the level of 40.05% without any other potentially toxic substances.

## Discussion

Carbon monoxide is extremely dangerous colorless, odorless, tasteless and non-irritating gas. These properties have earned it the title of “the silent killer”. Indeed, its sub-atmospheric density ensures invasion into space, particularly in confined areas, and explain hypoxic effect. For many years, the ability to block oxygen transport via its 250-fold greater affinity for hemoglobin constitutes the best known pathological effect. Then carboxyhemoglobin (COHb) levels measured in the CO-poisoned blood are formerly used as simplistic indicator of tissue hypoxia, to classify CO exposures in terms of mild (<30%), moderate (30-40%), or severe poisonings (>40%). Moreover, direct toxic effect of CO has been proved by inhibiting the activity of cytochrome-a3-oxidase and by causing lipid peroxidation. Hence, sufficient concentrations of CO in closed area may induce hypoxia and mitochondrial dysfunction leading to death within minutes [6].

CO intoxication represents a well-known method for committing suicide and has a long history. It has been mentioned, since 1844 by Eugene Sue in his book “The Wandering Jew”, where he noted the suicide of an old couple after dismissal from work [7]. In the early 20^th^ century, inhalation of domestic gas or car exhaust fumes was the most common method [8]. Along with the implementation of catalytic converters in motor vehicles and the decrease availability of CO in household gas, by improving gas installations and conversion to natural gas supply, the overall CO suicide rate has dropped [8,9]. Other new variants have emerged ever since, and there may have been a resulting increase in alternative methods of CO suicide, such as CB in a confined space [6]. This method has witnessed a dramatic increase in several Asian countries in the early nineties [10]. For example, it accounted only for 1.7% of Hong Kong suicides in 1998 and has jumped to 10.1% in 1999 [11]. Currently, it is considered as one of the most common suicide methods in Asia, reaching a rate of 30% of suicides in Taiwan, 10 to 25% in Japan and Hong Kong [12,13]. As well as increases of CB suicide was noted in South Korea, Singapore and Iran [14] and progressively this method has spread to the western countries [15-17]. However, the true incidence of CB-suicide all over the world is still incompletely known and many cases may be probably unrecognized [18]. To the best of our knowledge, this report is the first documented case of suicide by CB in Tunisia and probably in all African countries.

Anyway, publishing this type of manuscript is of paramount importance in sharing scientific knowledge, yet it is necessary to exercise caution and assess the magnitude of its risks. In 2014, World Health Organization (WHO) announced the suicide prevention strategy as a global state of emergency and highlighted the effect of inappropriate sharing of suicide cases on increasing the risk of imitation among vulnerable people. This mainly concerns internet and media suicide coverage when it comes to celebrities, as some outlets and platforms tend to report unusual methods, show images or provide information about the methods used or normalize suicide as an acceptable reaction to social crises [19]. Likewise, the Asian epidemic of CB suicide, in the 1990´s, was triggered in a very comparable context. Hong Kong was suffering from an economic depression in 1998, and suicide in general was increasing. After a local media massive publication of woman suicide inside her bedroom, CB had become the third major suicide method [20]. In 2003, the epidemic of CB suicide emerged in Japan. Stranger´s man and two women have formed a pact and suicide by CB in confined space. This incident drew significant media attention and initiated various cases of copycat suicide pacts in the following months [20]. Accordingly, several factors may have caused and promoted the rapid emergence and spread of CB suicides in Asian countries. Among, the description of this method as “soft” and “painless” by media and internet users played a key role [17]. In England, Chen *et al*. found that over one-third of these individuals had obtained information on suicide method from the internet [15]. Obviously, it has been argued that the internet may have a direct influence on suicide, since websites and chat rooms provide detailed information on suicide methods [5].

In our case, the confrontation of findings from the scene inspection, the police investigation, as well as autopsy and toxicological analysis results allow to determine the cause and manner of death. Firstly, careful examination of the scene highlights the planned character of death and suggests that potential involvement of third parties was unlikely. Secondly, post-mortem examination showed that cherry red livor mortis was matching the position of the corpse found in the scene. It also eliminated traumatic injuries and found black soot in the airways testifying that the deceased had breathed in the bathroom. Indeed, blackish incrustations under the fingernails indicated the charcoal manipulation by the deceased. Finally, toxicological analysis, carried out 7 days after the corpse discovery, found a relatively high COHb level. As, it is well known that COHb level decreases quickly after death, it is important to signal that COHb level in our case may be underestimated because of the time elapsed between death, autopsy and analysis [1,8]. Although, serious toxicity may occur when COHb level is greater than 25% notwithstanding that no dose-effect relationship was proved between COHb levels and the observed clinical effects within surviving victims [1]. Moreover, it was observed that victim of suicide by CO poisoning may use alcohol or hypnotic drugs [4]. Also, choosing CB as a suicide method has been associated with mental disorders, particularly severe depression, history of previous self-harm and earlier contact with psychiatric services [15]. It was also noted that 12% of CB suicide victims were suffering from serious relationship problems including divorce or emotional failure [15].

## Conclusion

Charcoal burning in a confined space is a duly old but renewed suicide method. In this paper, we report the first documented case of suicide by CB in Tunisia and probably in all African countries. Elsewhere, it is being commonly used as a suicide method in many countries not only due to the CO lethality but also to the faulty perception, wrought by media and internet users, of a relatively painless death. Therefore, all stakeholders including media professionals, website owners, and social networks users should not be only mindful of the potential negative impact of reporting unusual suicide cases, but also of their potential role in suicide prevention through responsible reporting. It is extremely important to stress this issue and prevent this method of suicide from mobilizing a hidden group of potential victims mainly in developing countries where socio-economic conditions may fuel its spread.
